# 3,3′-Dibenzoyl-1,1′-(butane-1,4-diyl)­dithio­urea

**DOI:** 10.1107/S1600536808004340

**Published:** 2008-03-05

**Authors:** Yu-Jie Ding, Xi-Bin Chang, Xiao-Qing Yang, Wen-Kui Dong

**Affiliations:** a Department of Biochemical Engineering, Anhui University of Technology and Science, Wuhu 241000, People’s Republic of China; b Qinghai Saltlake Industry Group Limited Company, Technological Center of Chemical Engineering, Geermu 81600, People’s Republic of China; c School of Chemical and Biological Engineering, Lanzhou Jiaotong University, Lanzhou 730070, People’s Republic of China

## Abstract

In the centrosymmetric title compound, C_20_H_22_N_4_O_2_S_2_, the carbonyl group forms an intra­molecular hydrogen bond with the NH group attached to the butanediyl linker, resulting in a six-membered ring. There are also inter­molecular C—H⋯S inter­actions in the crystal structure, and π–π inter­actions between phenyl groups [2.425 (3) Å].

## Related literature

For related literature, see: Breuzard *et al.* (2000 ▶); Burrows *et al.* (1997 ▶); Dong *et al.* (2006 ▶); Foss *et al.* (2004 ▶); Huang *et al.*, 2006 ▶; Nan *et al.* (2000 ▶); Teoh *et al.* (1999 ▶); Valdés-Martínez *et al.* (2004 ▶); Zhang *et al.* (2006 ▶).
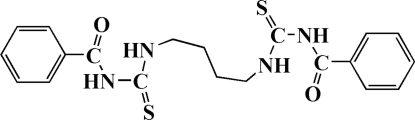


## Experimental

### 

#### Crystal data

C_20_H_22_N_4_O_2_S_2_
*M* *_r_* = 414.54
Monoclinic, 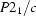

*a* = 6.0405 (11) Å
*b* = 23.358 (2) Å
*c* = 7.2877 (13) Å
β = 104.018 (2)°
*V* = 997.6 (3) Å^3^
*Z* = 2
Mo *K*α radiation
μ = 0.29 mm^−1^
*T* = 298 (2) K
0.22 × 0.16 × 0.07 mm

#### Data collection

Bruker SMART CCD area-detector diffractometer
Absorption correction: multi-scan (*SADABS*; Sheldrick, 1996 ▶) *T*_min_ = 0.939, *T*_max_ = 0.980
4845 measured reflections
1735 independent reflections
1044 reflections with *I* > 2σ(*I*)
*R*_int_ = 0.058

#### Refinement

*R*[*F* ^2^ > 2σ(*F* ^2^)] = 0.056
*wR*(*F* ^2^) = 0.106
*S* = 1.02
1735 reflections
127 parameters
H-atom parameters constrained
Δρ_max_ = 0.23 e Å^−3^
Δρ_min_ = −0.21 e Å^−3^

### 

Data collection: *SMART* (Siemens, 1996 ▶); cell refinement: *SAINT* (Siemens, 1996 ▶); data reduction: *SAINT*; program(s) used to solve structure: *SHELXS97* (Sheldrick, 2008 ▶); program(s) used to refine structure: *SHELXL97* (Sheldrick, 2008 ▶); molecular graphics: *SHELXTL* (Version 5.1; Sheldrick, 2008 ▶); software used to prepare material for publication: *SHELXTL*.

## Supplementary Material

Crystal structure: contains datablocks global, I. DOI: 10.1107/S1600536808004340/hg2377sup1.cif

Structure factors: contains datablocks I. DOI: 10.1107/S1600536808004340/hg2377Isup2.hkl

Additional supplementary materials:  crystallographic information; 3D view; checkCIF report

## Figures and Tables

**Table 1:** Hydrogen-bond geometry (Å, °)

*D*—H⋯*A*	*D*—H	H⋯*A*	*D*⋯*A*	*D*—H⋯*A*
N2—H2⋯O1	0.86	2.06	2.717 (3)	133
C2—H2*A*⋯S1	0.97	2.68	3.060 (3)	103
C2—H2*B*⋯S1^i^	0.97	2.72	3.468 (3)	134

Symmetry code: (i) 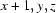.

**Fig. 1.**
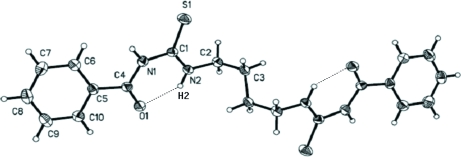
The molecular structure of (I)

## supplementary crystallographic information

### Comment

Acylthioureas have been the subject of extensive investigation because of their biological activity and their ability to coordinate strongly with metal ions (Teoh *et al.*, 1999; Huang *et al.*, 2006; Foss *et al.*, 2004). Some thioureas are organic catalysts in the metal-catalyzed asymmetric reduction of carbonyl compounds and carbonylative cyclization of *o*-hydroxyarylacetylenes (Nan *et al.*, 2000; Breuzard *et al.*, 2000). In recent years, thiourea derivatives have been studied because they are excellent H bonding donors and acceptors (Zhang *et al.*, 2006; Valdés-Martínez *et al.*, 2004), and readily form an intramolecular hydrogen bonding between the benzoyl (CO) and the N—H group (Dong *et al.*, 2006). They also easily form intermolecular hydrogen bonds, which can be applied in the design and synthesis of three-dimension supramolecular structure (Burrows *et al.*, 1997). Here we report synthesis and crystal structure of N, N'-(1, 4-tetramethylene)bisbenzoylthiourea (I), C_20_H_22_N_4_O_2_S_2_.

The crystal structure of (I) consists of discrete molecules. The carbonyl group forms an intramolecular hydrogen bond with the N2—H2 group, which forms a six-membered ring (C4/N1/C1/N2/H2/O1) structure, the H2···O1 bond length is 2.055 (3) Å. This is similar to the situation found in the structure of *N*-benzoyl-*N*'-(3-pyridyl)thiourea (Dong *et al.*, 2006). There is intermolecular hydrogen bonding between N2—H2 and the C?S group of another molecule, the H2···S1(*x* + 1, *y*, *z*) bond length is 2.906 (3) Å. The C?O bond length of 1.223 (3) Å is longer than the average C?O bond length (1.200 Å), which is due to intramolecular hydrogen bonding. The torsion angles of C2—N2—C1—N1 and C2—N2—C1—S1 are 178.3 (2) and -0.9 (4)°. There are π–π interactions between phenyl groups in the crystal lattice.

### Experimental

Benzoyl chloride (1.41 g, 10 mmol) was reacted with ammonium thiocyanate (1.14 g, 15 mmol) in CH_2_Cl_2_ (25 ml) solution under solid–liquid phase transfer catalysis, using polyethylene glycol-400 (0.18 g) as the catalyst, to give the corresponding benzoyl isothiocyanate. Then a solution of 1,4-butylenediamine (0.40 g, 4.5 mmol) in CH_2_Cl_2_ (15 ml) was added dropwise to benzoyl isothiocyanate, to give the title compound. Yield, 81.8%. m.p. 196–198 °C. Anal. Calc. for C_20_H_22_N_4_O_2_S_2_ (%): C, 57.97; H, 5.31; N, 13.53. Found: C, 57.90; H, 5.45; N, 13.35. Selected IR data (cm^-1^, KBr pellet): 3416, 3222 (ν NH), 1672 (ν C?O), 1146 (ν C?S). ^1^H NMR (200 MHz, DMSO-d6, δ, p.p.m.): 1.71 (t, 4H, CH2); 3.69 (t, 4H, CH2); 7.48–7.93 (m, 10H, C6H5); 10.95 (s, 1H, NH); 11.06 (s, 1H, NH). A DMF solution of the title compound was placed in a diethyl ether atmosphere, after several days, along with diffusion of diethyl ether into the DMF solution of the title compound, colorless block-shaped single crystals suitable for X-ray crystallographic analysis were obtained.

### Refinement

Non-H atoms were refined anisotropically. H atoms were treated as riding atoms with distances C—H = 0.97 (CH_2_), or 0.93 Å (CH), N—H = 0.86 Å, and *U*_iso_(H) = 1.2*U*_eq_(C) and 1.5*U*_eq_(N).

### Crystal data

**Table:**

C_20_H_22_N_4_O_2_S_2_	*F*_000_ = 436
*M**_r_* = 414.54	*D*_x_ = 1.380 Mg m^−^^3^
Monoclinic, *P*2_1_/*c*	Melting point = 469–471 K
Hall symbol: -P 2ybc	Mo *K*α radiation λ = 0.71073 Å
*a* = 6.0405 (11) Å	Cell parameters from 1545 reflections
*b* = 23.358 (2) Å	θ = 2.9–27.5º
*c* = 7.2877 (13) Å	µ = 0.29 mm^−^^1^
β = 104.018 (2)º	*T* = 298 (2) K
*V* = 997.6 (3) Å^3^	Block, colourless
*Z* = 2	0.22 × 0.16 × 0.07 mm

### Data collection

**Table:**

Bruker SMART CCD area-detector diffractometer	1735 independent reflections
Radiation source: fine-focus sealed tube	1044 reflections with *I* > 2σ(*I*)
Monochromator: graphite	*R*_int_ = 0.058
*T* = 298(2) K	θ_max_ = 25.0º
φ and ω scans	θ_min_ = 1.7º
Absorption correction: multi-scan(SADABS; Sheldrick, 1996)	*h* = −7→7
*T*_min_ = 0.939, *T*_max_ = 0.980	*k* = −27→21
4845 measured reflections	*l* = −8→7

### Refinement

**Table:**

Refinement on *F*^2^	Secondary atom site location: difference Fourier map
Least-squares matrix: full	Hydrogen site location: inferred from neighbouring sites
*R*[*F*^2^ > 2σ(*F*^2^)] = 0.056	H-atom parameters constrained
*wR*(*F*^2^) = 0.106	*w* = 1/[σ^2^(*F*_o_^2^) + (0.0375*P*)^2^ + 0.093*P*] where *P* = (*F*_o_^2^ + 2*F*_c_^2^)/3
*S* = 1.02	(Δ/σ)_max_ < 0.001
1735 reflections	Δρ_max_ = 0.23 e Å^−^^3^
127 parameters	Δρ_min_ = −0.21 e Å^−^^3^
Primary atom site location: structure-invariant direct methods	Extinction correction: none

### Special details

**Table:**

Geometry. All e.s.d.'s (except the e.s.d. in the dihedral angle between two l.s. planes) are estimated using the full covariance matrix. The cell e.s.d.'s are taken into account individually in the estimation of e.s.d.'s in distances, angles and torsion angles; correlations between e.s.d.'s in cell parameters are only used when they are defined by crystal symmetry. An approximate (isotropic) treatment of cell e.s.d.'s is used for estimating e.s.d.'s involving l.s. planes.
Refinement. Refinement of *F*^2^ against ALL reflections. The weighted *R*-factor *wR* and goodness of fit *S* are based on *F*^2^, conventional *R*-factors *R* are based on *F*, with *F* set to zero for negative *F*^2^. The threshold expression of *F*^2^ > σ(*F*^2^) is used only for calculating *R*-factors(gt) *etc*. and is not relevant to the choice of reflections for refinement. *R*-factors based on *F*^2^ are statistically about twice as large as those based on *F*, and *R*- factors based on ALL data will be even larger.

### Fractional atomic coordinates and isotropic or equivalent isotropic displacement parameters (Å^2^)

**Table:**

	*x*	*y*	*z*	*U*_iso_*/*U*_eq_	
N1	0.6333 (4)	0.33193 (10)	0.2464 (3)	0.0436 (7)	
H1	0.4996	0.3174	0.2342	0.052*	
N2	0.8393 (4)	0.41571 (9)	0.2530 (3)	0.0378 (6)	
H2	0.9588	0.3944	0.2747	0.045*	
O1	1.0080 (4)	0.30751 (8)	0.3060 (3)	0.0556 (7)	
S1	0.39076 (13)	0.42652 (4)	0.19513 (14)	0.0579 (3)	
C1	0.6387 (5)	0.39150 (12)	0.2345 (4)	0.0367 (7)	
C2	0.8669 (5)	0.47772 (12)	0.2383 (4)	0.0403 (8)	
H2A	0.7351	0.4931	0.1482	0.048*	
H2B	1.0004	0.4853	0.1902	0.048*	
C3	0.8930 (4)	0.50817 (12)	0.4263 (4)	0.0415 (8)	
H3A	0.8946	0.5491	0.4051	0.050*	
H3B	0.7612	0.4996	0.4755	0.050*	
C4	0.8082 (5)	0.29268 (13)	0.2746 (4)	0.0395 (8)	
C5	0.7380 (5)	0.23119 (12)	0.2626 (4)	0.0383 (8)	
C6	0.5157 (6)	0.21226 (13)	0.1896 (5)	0.0530 (9)	
H6	0.3997	0.2387	0.1462	0.064*	
C7	0.4663 (6)	0.15458 (15)	0.1811 (5)	0.0617 (10)	
H7	0.3171	0.1425	0.1316	0.074*	
C8	0.6341 (6)	0.11496 (14)	0.2445 (5)	0.0557 (10)	
H8	0.5993	0.0761	0.2394	0.067*	
C9	0.8531 (6)	0.13299 (14)	0.3154 (5)	0.0555 (9)	
H9	0.9681	0.1062	0.3581	0.067*	
C10	0.9057 (5)	0.19086 (13)	0.3241 (4)	0.0462 (9)	
H10	1.0558	0.2026	0.3720	0.055*	

### Atomic displacement parameters (Å^2^)

**Table:**

	*U*^11^	*U*^22^	*U*^33^	*U*^12^	*U*^13^	*U*^23^
N1	0.0352 (14)	0.0346 (15)	0.060 (2)	−0.0048 (12)	0.0101 (13)	−0.0022 (13)
N2	0.0309 (14)	0.0340 (14)	0.0476 (17)	0.0051 (11)	0.0077 (12)	−0.0024 (12)
O1	0.0374 (13)	0.0408 (13)	0.0810 (18)	−0.0008 (10)	−0.0005 (12)	0.0001 (12)
S1	0.0363 (5)	0.0507 (5)	0.0883 (8)	0.0062 (4)	0.0181 (5)	0.0070 (5)
C1	0.0325 (17)	0.0413 (18)	0.036 (2)	0.0030 (14)	0.0078 (14)	0.0004 (15)
C2	0.0375 (17)	0.0373 (18)	0.047 (2)	0.0039 (14)	0.0125 (15)	0.0036 (16)
C3	0.0386 (17)	0.0299 (17)	0.055 (2)	0.0042 (13)	0.0102 (16)	0.0017 (16)
C4	0.043 (2)	0.0399 (19)	0.033 (2)	0.0048 (15)	0.0035 (16)	−0.0001 (15)
C5	0.0455 (19)	0.0358 (18)	0.034 (2)	0.0012 (15)	0.0108 (16)	−0.0009 (15)
C6	0.047 (2)	0.042 (2)	0.065 (3)	0.0019 (16)	0.0047 (18)	0.0009 (19)
C7	0.056 (2)	0.049 (2)	0.075 (3)	−0.0142 (18)	0.007 (2)	−0.005 (2)
C8	0.074 (3)	0.039 (2)	0.057 (3)	−0.0035 (19)	0.021 (2)	0.0001 (18)
C9	0.071 (3)	0.043 (2)	0.054 (3)	0.0150 (19)	0.018 (2)	0.0049 (18)
C10	0.044 (2)	0.044 (2)	0.048 (2)	0.0045 (16)	0.0070 (17)	−0.0015 (17)

### Geometric parameters (Å, °)

**Table:**

N1—C4	1.376 (3)	C3—H3B	0.9700
N1—C1	1.395 (3)	C4—C5	1.494 (4)
N1—H1	0.8600	C5—C10	1.376 (4)
N2—C1	1.314 (3)	C5—C6	1.391 (4)
N2—C2	1.465 (3)	C6—C7	1.378 (4)
N2—H2	0.8600	C6—H6	0.9300
O1—C4	1.223 (3)	C7—C8	1.368 (4)
S1—C1	1.669 (3)	C7—H7	0.9300
C2—C3	1.518 (4)	C8—C9	1.365 (4)
C2—H2A	0.9700	C8—H8	0.9300
C2—H2B	0.9700	C9—C10	1.387 (4)
C3—C3^i^	1.516 (5)	C9—H9	0.9300
C3—H3A	0.9700	C10—H10	0.9300
			
C4—N1—C1	130.2 (2)	O1—C4—N1	121.8 (3)
C4—N1—H1	114.9	O1—C4—C5	122.4 (3)
C1—N1—H1	114.9	N1—C4—C5	115.8 (3)
C1—N2—C2	122.4 (2)	C10—C5—C6	118.2 (3)
C1—N2—H2	118.8	C10—C5—C4	117.6 (3)
C2—N2—H2	118.8	C6—C5—C4	124.2 (3)
N2—C1—N1	117.2 (2)	C7—C6—C5	120.4 (3)
N2—C1—S1	125.0 (2)	C7—C6—H6	119.8
N1—C1—S1	117.8 (2)	C5—C6—H6	119.8
N2—C2—C3	112.7 (2)	C8—C7—C6	120.8 (3)
N2—C2—H2A	109.1	C8—C7—H7	119.6
C3—C2—H2A	109.1	C6—C7—H7	119.6
N2—C2—H2B	109.1	C9—C8—C7	119.3 (3)
C3—C2—H2B	109.1	C9—C8—H8	120.3
H2A—C2—H2B	107.8	C7—C8—H8	120.3
C3^i^—C3—C2	114.0 (3)	C8—C9—C10	120.6 (3)
C3^i^—C3—H3A	108.8	C8—C9—H9	119.7
C2—C3—H3A	108.8	C10—C9—H9	119.7
C3^i^—C3—H3B	108.8	C5—C10—C9	120.7 (3)
C2—C3—H3B	108.8	C5—C10—H10	119.7
H3A—C3—H3B	107.7	C9—C10—H10	119.7
			
C2—N2—C1—N1	178.3 (2)	O1—C4—C5—C6	−166.1 (3)
C2—N2—C1—S1	−0.9 (4)	N1—C4—C5—C6	13.4 (4)
C4—N1—C1—N2	−0.3 (4)	C10—C5—C6—C7	0.5 (5)
C4—N1—C1—S1	179.0 (2)	C4—C5—C6—C7	179.0 (3)
C1—N2—C2—C3	88.5 (3)	C5—C6—C7—C8	0.2 (5)
N2—C2—C3—C3^i^	64.4 (4)	C6—C7—C8—C9	−0.6 (5)
C1—N1—C4—O1	4.7 (5)	C7—C8—C9—C10	0.3 (5)
C1—N1—C4—C5	−174.8 (3)	C6—C5—C10—C9	−0.8 (5)
O1—C4—C5—C10	12.4 (4)	C4—C5—C10—C9	−179.4 (3)
N1—C4—C5—C10	−168.1 (3)	C8—C9—C10—C5	0.4 (5)

Symmetry codes: (i) −*x*+2, −*y*+1, −*z*+1.

### Hydrogen-bond geometry (Å, °)

**Table:**

*D*—H···*A*	*D*—H	H···*A*	*D*···*A*	*D*—H···*A*
N2—H2···O1	0.86	2.06	2.717 (3)	133
C2—H2A···S1	0.97	2.68	3.060 (3)	103
C2—H2B···S1^ii^	0.97	2.72	3.468 (3)	134

Symmetry codes: (ii) *x*+1, *y*, *z*.
